# Silencing markers are retained on pericentric heterochromatin during murine primordial germ cell development

**DOI:** 10.1186/s13072-017-0119-3

**Published:** 2017-03-11

**Authors:** Aristea Magaraki, Godfried van der Heijden, Esther Sleddens-Linkels, Leonidas Magarakis, Wiggert A. van Cappellen, Antoine H. F. M. Peters, Joost Gribnau, Willy M. Baarends, Maureen Eijpe

**Affiliations:** 1 Department of Developmental Biology, Erasmus MC, University Medical Center, Rotterdam, The Netherlands; 2 Division of Reproductive Medicine, Department of Obstetrics and Gynecology, Erasmus MC, Rotterdam, The Netherlands; 3 Friedrich Miescher Institute for Biomedical Research (FMI), Basel, Switzerland; 4 Faculty of Sciences, University of Basel, Basel, Switzerland; 5 Division of Reproductive Medicine, Department of Obstetrics and Gynecology, Central Hospital of Karlstad, Karlstad, Värmland Sweden; 6 Erasmus Optical Imaging Center, Erasmus MC, Rotterdam, The Netherlands

**Keywords:** Pericentric heterochromatin, Primordial germ cell, Centromere, Histone modifications, H3K9me3, H4K20me3, ATRX, HP1, Immunochemistry, Major satellites

## Abstract

**Background:**

In the nuclei of most mammalian cells, pericentric heterochromatin is characterized by DNA methylation, histone modifications such as H3K9me3 and H4K20me3, and specific binding proteins like heterochromatin-binding protein 1 isoforms (HP1 isoforms). Maintenance of this specialized chromatin structure is of great importance for genome integrity and for the controlled repression of the repetitive elements within the pericentric DNA sequence. Here we have studied histone modifications at pericentric heterochromatin during primordial germ cell (PGC) development using different fixation conditions and fluorescent immunohistochemical and immunocytochemical protocols.

**Results:**

We observed that pericentric heterochromatin marks, such as H3K9me3, H4K20me3, and HP1 isoforms, were retained on pericentric heterochromatin throughout PGC development. However, the observed immunostaining patterns varied, depending on the fixation method, explaining previous findings of a general loss of pericentric heterochromatic features in PGCs. Also, in contrast to the general clustering of multiple pericentric regions and associated centromeres in DAPI-dense regions in somatic cells, the pericentric regions of PGCs were more frequently organized as individual entities. We also observed a transient enrichment of the chromatin remodeler ATRX in pericentric regions in embryonic day 11.5 (E11.5) PGCs. At this stage, a similar and low level of major satellite repeat RNA transcription was detected in both PGCs and somatic cells.

**Conclusions:**

These results indicate that in pericentric heterochromatin of mouse PGCs, only minor reductions in levels of some chromatin-associated proteins occur, in association with a transient increase in ATRX, between E11.5 and E13.5. These pericentric heterochromatin regions more frequently contain only a single centromere in PGCs compared to the surrounding soma, indicating a difference in overall organization, but there is no de-repression of major satellite transcription.

**Electronic supplementary material:**

The online version of this article (doi:10.1186/s13072-017-0119-3) contains supplementary material, which is available to authorized users.

## Background

Chromatin is composed of DNA, histones, and other tightly associated proteins. Modifications of the DNA and of histones directly or indirectly control the regulation of DNA-related processes like transcription. Globally, the chromatin in a nucleus can be functionally divided into active and accessible euchromatin and inactive and condensed heterochromatin. Heterochromatin exists in two forms: facultative and constitutive heterochromatin. Facultative heterochromatin is a flexible form of heterochromatin and can be found in various chromosomal regions, when gene-coding regions need to be repressed. Its size varies from gene clusters to an entire chromosome (the inactive *X* in female cells). Facultative heterochromatin is frequently marked by specific histone modifications such as H2AK119Ub and H3K27me3, mediated by the polycomb repressor complexes (PRC) 1 and 2, respectively. Constitutive heterochromatin forms at specific regions of the genome, which are characterized by arrays of tandem DNA repeats: at the centromeres (minor satellite repeats), telomeres (telomeric repeats), and pericentric regions (major satellite repeats). Here we focus on the pericentric heterochromatin. A known hallmark of this chromatin type is the lack of histone modifications that generally mark active chromatin, such as histone acetylation. Conversely, there is an accumulation of repressive histone marks such as H3K9me3 and H4K20me3 [1–5]. The presence of H3K9me3 results in recruitment of different heterochromatin protein (HP) isoforms that contribute to heterochromatin establishment and maintenance of this chromatin state [6, 7]. The basic unit of the major satellites in the mouse is an A/T-rich ~230-bp-long monomer, which can be repeated many times, leading to regions of up to several megabases in size. In an interphase mouse nucleus, pericentric constitutive heterochromatin can be visualized as 4′,6-diamidino-2-phenylindole (DAPI)-dense regions, termed chromocenters, with each chromocenter consisting of multiple pericentric regions from different chromosomes. The periphery of each chromocenter contains the centromeres of the chromosomes as individual entities [8].

Maintenance of the heterochromatic nature of pericentric DNA is important for proper cell functions; failure impairs cell viability, induces chromosomal instabilities, and increases the risk of tumorigenesis [2]. Therefore, pericentric heterochromatin has for a long time been considered as an inert, highly condensed, and inaccessible domain. In recent years, however, it has become clear that the biology of pericentric heterochromatin is more complicated. Emerging evidence indicates that some well-controlled dynamical changes of pericentric heterochromatin structure may occur, which are associated in some cases with brief bursts of major satellite transcription. Transcription of major satellites has been shown to occur during canonical cell processes, e.g. during the normal cell cycle [9, 10], cell differentiation [11, 12], and during early [13, 14] and late [15] embryonic development. For example, in pre-implantation mouse embryos, the paternal pericentric domains initially lack heterochromatin marks, such as H3K9me3 and HP1 proteins. This likely relates to the fact that the paternal genome enters the oocyte as a protamine-packaged compact structure, largely devoid of nucleosomes. After fertilization, the DNA rapidly decondenses as protamines are removed and replaced by maternal histones that lack pericentric heterochromatin histone modifications [16–19]. Concomitantly, active DNA demethylation occurs [16, 20]. In contrast, maternal pericentric heterochromatin displays the typical somatic histone posttranslational modification marks. Interestingly, major satellites are transcribed (in forward direction) solely from the paternal pronucleus at the 2-cell stage, which might reflect the above-described specific epigenetic status of the paternal genome [21]. Then, a burst in transcription of the major satellites (in reversed direction) from both parental genomes facilitates the reorganization of pericentric heterochromatin from nuclear precursor bodies to the typical somatic like chromocenters in the developing embryo. This is completed by the 4- to 8-cell stage after which pericentric heterochromatin displays its specific H3K9me3–HP1 chromatin state [14, 22].

Developing mouse primordial germ cells (PGCs) also undergo genome-wide epigenetic reprogramming, and this occurs between E8.0 and E13.5. It includes changes in histone modifications (e.g. global loss of H3K9me2 and relative enrichment of H3K27me3 compared to somatic cells as assessed by immunofluorescence experiments), reactivation of the inactive *X* chromosome in the female embryos, and global loss of DNA methylation, the last reaching its lowest levels at E13.5, both in male and female embryos [23, 24].

Initiation of imprint erasure in PGCs takes place between E10.5 and E11.5 [25, 26], and concomitantly, it has been reported that PGCs lose the DAPI-dense chromocenters [25]. These events are accompanied by a transient apparent loss of H3K9me3, HP1 proteins, and other heterochromatin marks [27]. In this study, we focus specifically on the pericentric heterochromatin in germ cells between E10.5 and E13.5 of mouse embryo development. Since we experienced difficulties to reproduce the previously reported transient loss of pericentric heterochromatin marks [27], we decided to revisit the possible loss and re-establishment of pericentric heterochromatin marks and of chromocenters during PGC development, by testing different preparation methods and fixation conditions. It is well known that different fixation and preparation methods may lead to variations in immunostaining results, and these should thus be interpreted with caution. In particular, the inability to detect a protein does not always result from its absence, but could be caused, for example, by epitope masking. Using a method that is known as “drying-down” or “spreading” of (meiotic) nuclei [28], we observed persistence of H3K9me3, HP1 isoforms, and H4K20me3 on pericentric heterochromatin of PGCs. Based on these results, we conclude that the reported loss and re-establishment of pericentric heterochromatin signature [27] may reflect a structural change in pericentric heterochromatin, affecting epitope availability, rather than the actual loss of the markers. In addition, we found ATRX, a chromatin remodeler known to associate with constitutive heterochromatin [29, 30], to be highly enriched at pericentric heterochromatin in PGCs at E11.5 compared to the somatic cells of the same developmental stage. Lastly, immunofluorescent analysis of centromere and pericentromere (adjacent to the centromeres) staining showed that pericentromeres do not cluster together in the same fashion as in the surrounding somatic cells, and this may explain the weak DAPI staining of pericentric heterochromatin in developing PGCs. Still, consistent with the overall persistence of histone modifications and the enrichment of ATRX, no increased transcription of major satellite repeats was detected in isolated E11.5 PGCs. Together, our data indicate that although the pericentric heterochromatin in E11.5 mouse PGCs may exist in a different chromatin conformation and is organized more frequently as small regions containing a single centromere compared to somatic cells, this phenomenon is neither associated with a complete loss of heterochromatin hallmarks nor with a burst in transcription of major satellite repeats.

## Results

### H3K9me3 remains present in pericentric heterochromatin throughout germ cell development

**Fig. 1:**
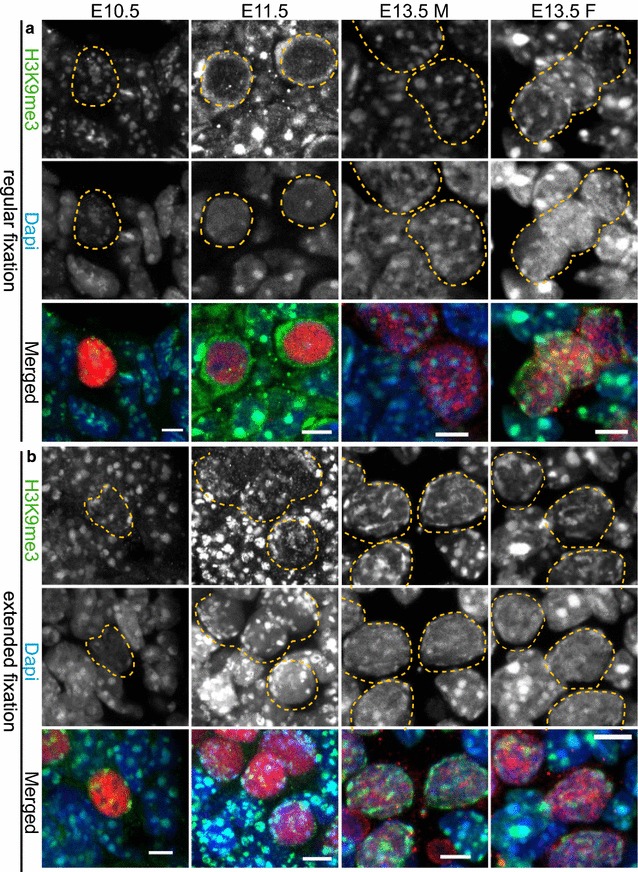
H3K9me3 signal enrichment persists on pericentric heterochromatin throughout germ cell development upon extended tissue fixation. **a** Immunofluorescent analysis of H3K9me3 (*green*) in cryosections of E10.5 and E11.5 trunks containing germ cells and of E13.5 male and female gonads using the regular fixation protocol. H3K9me3 staining is present in DAPI (*blue*)-dense regions of E10.5 PGCs and somatic cells. At E11.5 H3K9me3 transiently disappears from pericentric heterochromatin of E11.5 PGCs only. Thereafter, H3K9me3 returns in E13.5 DAPI-dense regions. **b** Using extended fixation, H3K9me3 is retained in DAPI-dense regions of PGCs and somatic cells in all embryonic stages examined. E10.5 and E11.5 germ cells are marked with OCT4 (*red*), while E13.5 germ cells are marked with TRA98 (*red*). Using regular or extended fixation, two embryos or gonads were analysed per stage, and at least 20 PGC nuclei were recorded. Representative germ cells are marked with *yellow dashed circles*. *Scale bars* represent 5 μm

**Fig. 2:**
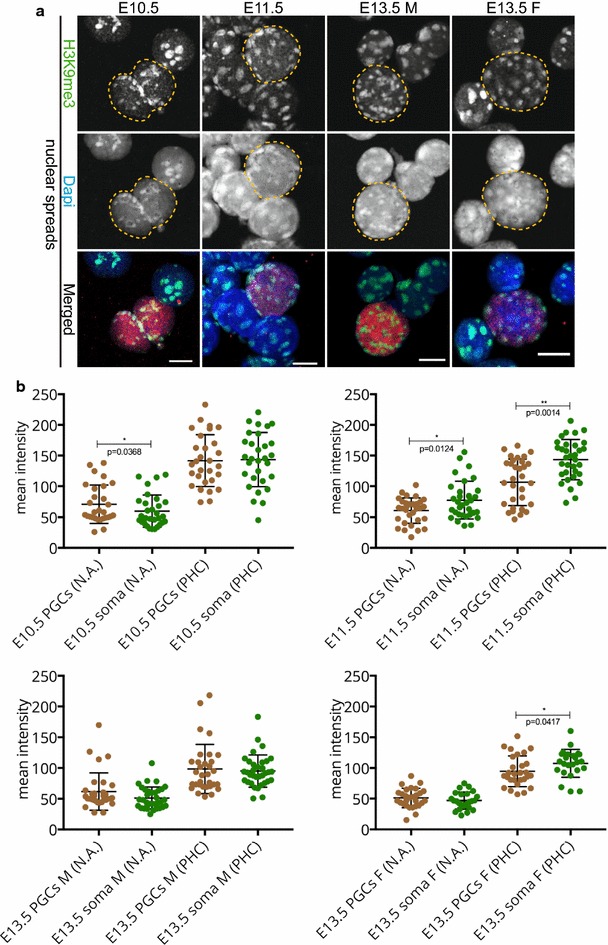
H3K9me3 signal enrichment persists on pericentric heterochromatin throughout germ cell development in spread preparations. **a** Spread preparations immunostained for H3K9me3 (*green*). E10.5 and E11.5 germ cells are marked with OCT4 (*red*), while E13.5 germ cells are marked with TRA98 (*red*). Representative germ cells are marked with *yellow dashed circles*. *Scale bars* represent 10 μm. **b** Quantification analysis of mean H3K9me3 levels in the whole nuclear area (N.A.) and pericentric heterochromatin regions (PHC) in the different embryonic stages examined. Four or more embryo trunks or gonads were pooled for the nuclear spread preparations and 20–30 PGC, and somatic nuclei were recorded. *Asterisks* (* or **) indicate significant differences

We first reanalysed the reported dynamics of H3K9me3 [27] in PGCs of E10.5–E13.5 mouse embryos. For this, we used a fluorescent immunohistochemical approach. Since fixation conditions may influence epitope availability, we fixed and embedded embryos using different protocols. OCT4 (E10.5 and E11.5) or TRA98 (E13.5) was used as germ cell markers. For H3K9me3 staining, we did not observe any robust and reproducible staining pattern using paraffin-embedded tissue sections. In contrast, cryosectioning of paraformaldehyde-fixed samples did produce the typical pattern of H3K9me3 enrichment in heterochromatin areas of somatic cells. Interestingly, using two different fixation protocols, one involving fixation only prior to freezing and embedding (regular fixation), and another protocol that included a postfixation step after sectioning (extended fixation, see Methods for details), two different staining patterns were observed. Using regular or extended fixation, two embryos or gonads were analysed for each studied protein or histone modification per stage, and qualitative analyses for the patterns in PGC nuclei compared to surrounding somatic cells were recorded for at least 20 PGCs. Using both methods, the pattern of H3K9me3 immunostaining in PGCs was similar to that of surrounding somatic cells at E10.5, despite the overall DAPI weak appearance of the PGC chromocenters (Fig. 1, panel a and b). Using the regular fixation procedure, we observed an overall reduction of H3K9me3 signal solely from E11.5 germ cells, in accordance with previous observations [27] (Fig. 1, panel a). In contrast, when using extended fixation, H3K9me3 signal was retained on the pericentric heterochromatin as the PGCs developed between E10.5 and E13.5 (Fig. 1, panel b). As an alternative approach, and to further ensure epitope availability, we used a drying-down alias meiotic spread method that is commonly used to study the localization of chromatin modifications and associated proteins in nuclei of meiotic prophase cells [28]. It involves mixing of a cell suspension on a glass slide covered with a Triton X100-containing fixative, followed by gradual drying, whereby the nuclei spread on glass. The spreading results in loss of most cytoplasmic and loosely DNA-associated proteins and flattening of the nuclear chromatin. Therefore, we will further refer to this type of preparation as nuclear spreads. We prepared slides containing nuclei from E10.5 to E11.5 gonadal regions and from E13.5 male and female gonads. Since these preparations are relatively flat, and a single plane image at optimal focus provides a reproducible estimate for the amount of signal, we decided to include a quantitative analysis with this approach (see Methods for details concerning the quantification method). Similar to the results obtained with the extended fixation protocol, H3K9me3 signal was retained in the pericentric heterochromatin of PGCs from E10.5 to E13.5, although the signals are reduced in pericentric heterochromatin of E11.5 PGCs compared to pericentric heterochromatin of the soma (Fig. 2a, b). This indicates that there may be some difference between the pericentric heterochromatin structure of PGCs and somatic cells, but there is no major loss of H3K9me3 from the pericentric regions in E11.5 PGCs. In accordance with Kagiwada et al. [26], we did not observe complete loss of the DAPI-dense regions at any of the examined stages in all protocols tested, but the regions appeared less DAPI intense and at the same time smaller. Importantly, our results indicate that the previously reported absence of pericentric heterochromatin marks in E11.5 PGCs might be a consequence of the chosen experimental methodology.

### HP1 isoforms are stably recruited to pericentric heterochromatin of developing germ cells

**Fig. 3:**
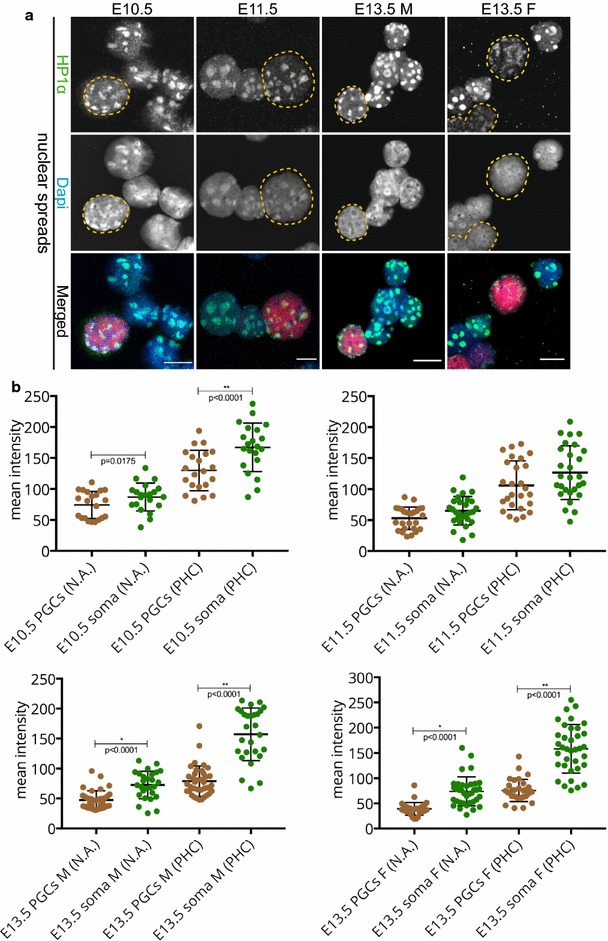
HP1α is recruited to pericentric heterochromatin in E10.5–E13.5 germ cells. **a** Nuclear spread preparations from E10.5 to E11.5 embryos and from E13.5 male and female gonads were stained for HP1α (*green*). The pericentric heterochromatin of germ cells was always decorated with HP1α. E10.5 and E11.5 germ cells are marked with OCT4 (*red*), while E13.5 germ cells are marked with TRA98 (*red*). Four or more embryo trunks or gonads were pooled for the nuclear spread preparations and 20–30 PGC, and somatic nuclei were recorded. Representative germ cells are marked with *yellow dashed circles*. *Scale bars* represent 10 μm. **b** Quantification analysis of HP1α levels in the whole nuclear area (N.A.) and pericentric heterochromatin regions (PHC) in the different embryonic stages examined. *Asterisks* (* or **) indicate significant differences

Specific histone modifications recruit certain proteins. Members of the heterochromatin protein 1 (HP1) protein family bind H3K9me3 and mark constitutive heterochromatin. In mammals, there are three different HP1 isoforms: HP1α, HP1β, and HP1γ also known as CBX5, CBX1, and CBX3, respectively. We examined the localization of these three isoforms during PGC development. In the regular fixation protocol and using paraffin sections, HP1α immunostaining marked pericentric heterochromatin in E10.5 PGCs. Already at this stage, the signal for HP1α appeared lower in PGCs compared to surrounding somatic cells. Thereafter, HP1α was undetectable in developing germ cells (Additional file 1, panel A), which is in accordance with previous studies [27]. Using extended fixation conditions, HP1α signal was reduced (E10.5, E11.5 some PGCs, E13.5 female germ cells) or absent (E11.5 some PGCs, E13.5 male germ cells). It should be noted that in E13.5 male gonad sections, we could not reproducibly detect HP1α even in the surrounding somatic cells of sections, using either regular or extended fixation protocols (Additional file 1, panel A and B). This may be due to the different consistency of the male versus the female gonad at this age, causing differential and variable effects of the fixation protocols. Specifically, we observe that at E13.5 the ovarian tissue is softer and more vulnerable to dissociation compared to the developing testis, which seems more compact. When nuclear spreads from genital ridges or embryonic gonads were examined, HP1α immunostaining was readily detectable and enriched in DAPI-dense regions of all cells, but this enrichment was more clear in the pericentric heterochromatin areas of the soma compared to those of the corresponding PGCs in all developmental stages examined, but most clearly at E13.5 (Fig. 3a, b).

**Fig. 4:**
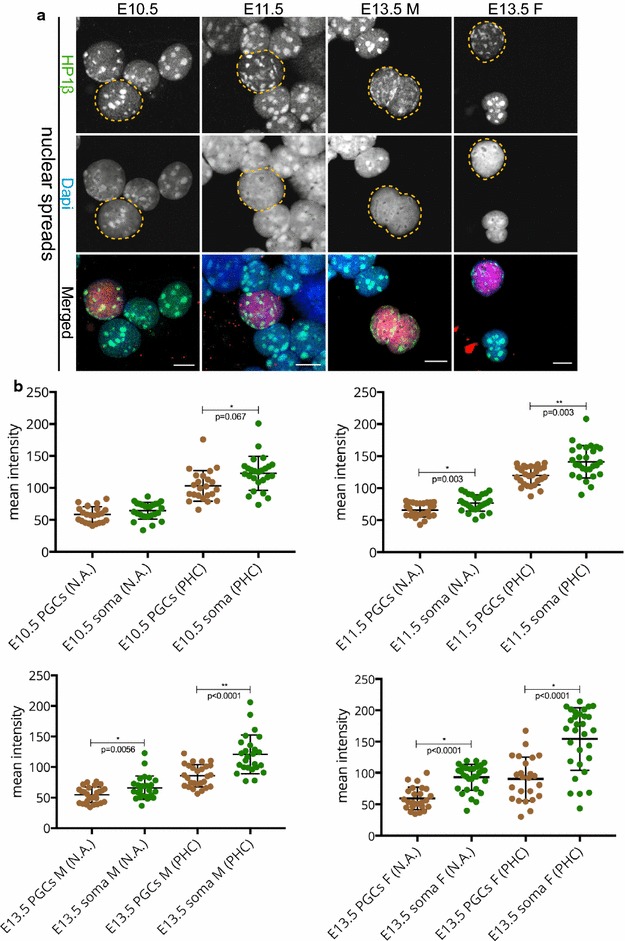
HP1β is recruited to pericentric heterochromatin in E10.5–E13.5 germ cells. **a** Nuclear spread preparations from E10.5 to E11.5 embryos and from E13.5 male and female gonads were stained for HP1β (*green*). This protein was always present in DAPI (*blue*)-dense regions of germ cells in all stages examined. E10.5 and E11.5 germ cells are marked with OCT4 (*red*), while E13.5 germ cells are marked with TRA98 (*red*). Four or more embryo trunks or gonads were pooled for the nuclear spread preparations and 20–30 PGC, and somatic nuclei were recorded. Representative germ cells are marked with *yellow dashed circles*. *Scale bars* represent 10 μm. **b** Quantification analysis of HP1β levels in the whole nuclear area (N.A.) and pericentric heterochromatin regions (PHC) in the different embryonic stages examined. *Asterisks* (* or **) indicate significant differences

Like HP1α, HP1β is also known to predominantly localize to heterochromatin. When we use our regular fixation protocol, we observed accumulation of HP1β signal in DAPI-dense regions of both somatic and germ cell nuclei at E10.5 and E11.5 stages. However, at E13.5, hardly any enrichment was observed in the DAPI-dense regions of both male and female germ cells (Additional file 2, panel A). Following extended fixation, HP1β signal was preserved on pericentric heterochromatin in all developmental germ cell stages examined (Additional file 2, panel B), similar to what was observed in nuclear spread preparations (Fig. 4a). The quantitative measurements showed (similar to HP1α) that HP1β is present at a higher level in the pericentric heterochromatin of the soma compared to that of the PGCs (Fig. 4b).

**Fig. 5:**
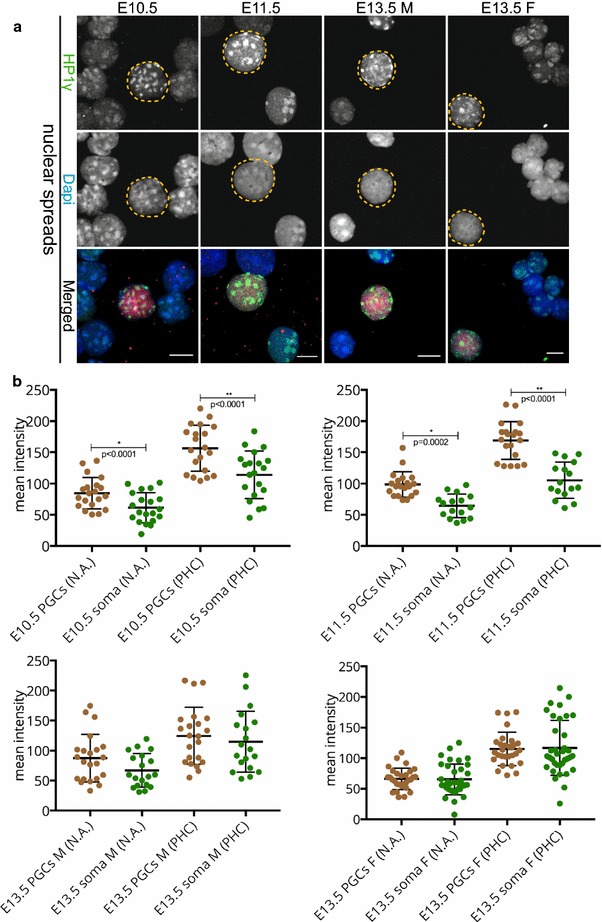
HP1γ is recruited to pericentric heterochromatin in E10.5–E13.5 germ cells. **a** Nuclear spread preparations from E10.5 to E11.5 embryos and from E13.5 male and female gonads were stained for HP1γ (*green*). This protein is also stably recruited to pericentric heterochromatin of PGCs, in the three stages examined. E10.5 and E11.5 germ cells are marked with OCT4 (*red*), while E13.5 germ cells are marked with TRA98 (*red*). Four or more embryo trunks or gonads were pooled for the nuclear spread preparations and 20–30 PGC, and somatic nuclei were recorded. Representative germ cells are marked with *yellow dashed circles*. *Scale bars* represent 10 μm. **b** Quantification analysis of HP1γ levels on the whole nuclear area (N.A.) and pericentric heterochromatin regions (PHC) in the different embryonic stages examined. The levels of HP1γ isoform at E10.5 and E11.5 in the pericentric heterochromatin regions are notably more enriched compared to those of the corresponding somatic cells. *Asterisks* (* or **) indicate significant differences

The last HP1 isoform, HP1γ, is known to interact with both constitutive heterochromatin and euchromatin [31–33]. Examination of HP1γ in nuclear spreads revealed a clear immunostaining signal for this HP1 isoform in DAPI-dense regions in the nuclei of germ cells throughout development. Interestingly, HP1γ levels were significantly higher in the nuclei (and pericentric heterochromatin areas) of E10.5 and E11.5 PGCs, compared to the surrounding soma. At E13.5, HP1γ signals were similar in PGCs and somatic nuclei (Fig. 5a, b). Comparable results were obtained upon regular or extended fixation in paraffin sections of E10.5, E11.5 genital ridges, and E13.5 female gonads (Additional file 3). However, at E13.5 of male development, detection of HP1γ in the surrounding somatic cells of paraffin sections was difficult and variable, using either our regular or extended fixation protocol. This was similar to our HP1α results. We could detect accumulation of HP1γ in DAPI-dense regions in some E13.5 male germ cells (Additional file 3, panel A and B, arrowhead), but not in all.

Taken together, the persistent detection of the H3K9me3 on pericentric heterochromatin during PGC development is consistent with the patterns observed for the HP1 proteins, whereby decreases in HP1α and HP1β may be at least partially compensated by an increase in HP1γ.

### H4K20me3 is retained at pericentric heterochromatin of E11.5 PGCs

**Fig. 6:**
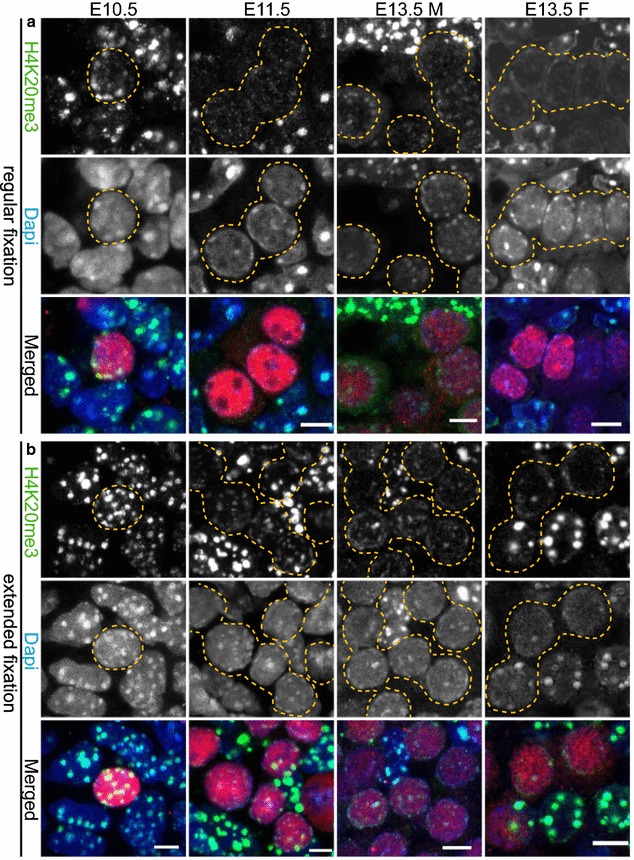
H4K20me3 enrichment is detected in pericentric heterochromatin of developing germ cells. **a** Paraffin sections of E10.5 and E11.5 embryo trunks and of E13.5 male and female gonads were immunostained using anti-H4K20me3 (*green*) after applying the regular fixation protocol. At E10.5 H4K20me3 is present in pericentric heterochromatin of PGCs and surrounding soma. At E11.5, H4K20me3 is lost from DAPI (*blue*)-dense regions of PGCs, while it is maintained in the somatic cells. At E13.5 H4K20me3 reappears in germ cells, but in substantially reduced levels compared to the surrounding gonadal somatic cells. **b** When applying the extended fixation protocol, H4K20me3 is retained at pericentric heterochromatin in PGC nuclei from E10.5 to E13.5. However, when compared to the H4K20me3 pattern in the surrounding somatic cells, the levels are reduced. E10.5 and E11.5 germ cells are marked with OCT4 (*red*), while E13.5 germ cells are marked with TRA98 (*red*). Using regular or extended fixation, two embryos or gonads were analysed per stage, and at least 20 PGC nuclei were recorded. Representative germ cells are marked with *yellow dashed circles*. *Scale bars* represent 5 μm

**Fig. 7:**
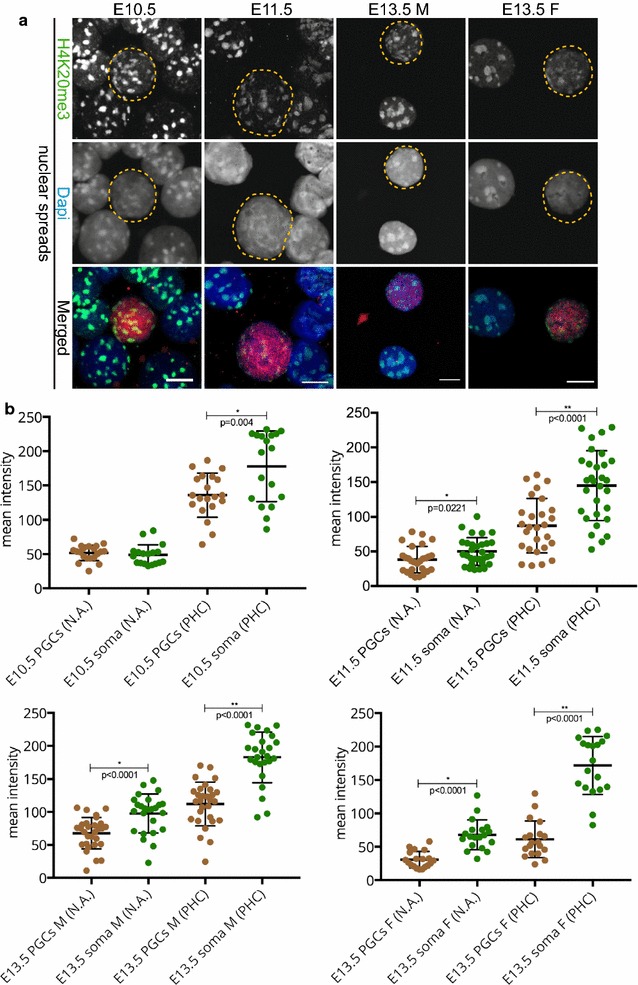
H4K20me3 enrichment is detected in pericentric heterochromatin of developing germ cells in spread preparations. **a** Nuclear spread preparations from E10.5 to E11.5 embryos and from E13.5 male and female gonads were stained for H4K20me3 (*green*). This modification is enriched at pericentric heterochromatin in PGCs throughout germ cell development when analysed. E10.5 and E11.5 germ cells were identified by OCT4 (*red*), and E13.5 germ cells with TRA98 (*red*). Three or more embryo trunks or gonads were pooled for the nuclear spread preparations and 20–30 PGC, and somatic nuclei were recorded. Representative germ cells are marked with *yellow dashed circles*. *Scale bars* represent 10 μm. **b** Quantification analysis of H4K20me3 levels in the whole nuclear area (N.A.) and pericentric heterochromatin regions (PHC) in the different embryonic stages examined. Overall, the levels of H4K20me3 are significantly reduced in PGC pericentric heterochromatin areas compared to those of the surrounding somatic cells. *Asterisks* (* or **) indicate significant differences

An additional histone mark that participates in the establishment of pericentric heterochromatin is H4K20me3 [4, 5]. This histone modification is mediated by the histone methyltransferase SUV4-20H2 in a SUV39H and HP1-dependent manner [4, 34]. SUV39H is the enzyme responsible for establishing trimethylation of H3K9 [2]. Similar to H3K9me3, H4K20me3 is strongly enriched at DAPI-dense regions [4, 5]. Again we performed comparative immunofluorescence in sections processed with regular and extended fixation, and the more quantitative analyses in nuclear spreads. When using the regular fixation procedure on paraffin-embedded embryo sections, H4K20me3 signal intensity was similar in developing PGCs and surrounding somatic cells at E10.5 (Fig. 6a). However, at E11.5, the immunostaining signal for this histone modification transiently disappeared from the DAPI-dense chromocenters of the PGCs only, while it was strongly retained in the surrounding somatic cells (Fig. 6a). Two days later, at ~E13.5, H4K20me3 signal reappeared, albeit at low levels compared to the surrounding soma and only in some TRA98 (red)-positive cells, regardless of the embryo sex. When using the extended fixation protocol, H4K20me3 signal was retained on pericentric heterochromatin throughout PGC development, but clearly reduced in the PGCs compared to the surrounding somatic cells at E11.5–E13.5 (Fig. 6b). Lastly, upon analysis of nuclear spread preparations, we indeed observed that H4K20me3 is significantly reduced in pericentric heterochromatin regions of PGCs compared to the soma in all stages examined (Fig. 7a, b). Importantly, similar to H3K9me3, H4K20me3 did not fully disappear from the DAPI-dense regions of E11.5 PGCs.

### ATRX is enriched in pericentric heterochromatin of primordial germ cells

**Fig. 8:**
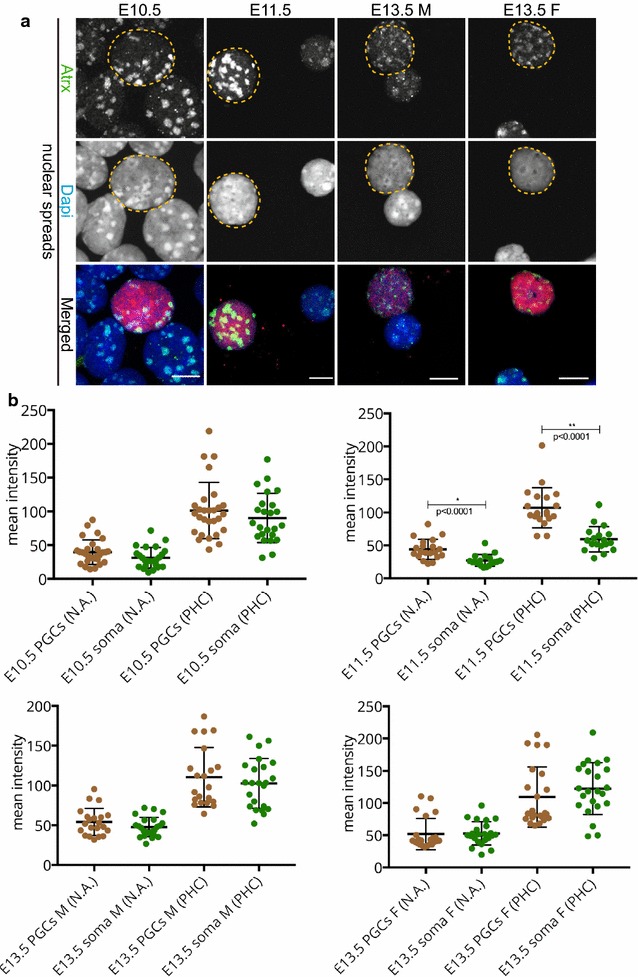
ATRX is more enriched at pericentric heterochromatin of E11.5 PGCs compared to that of the surrounding soma. **a** Analysis of ATRX (*green*) localization patterns in nuclear spread preparations of E10.5 and E11.5 embryo trunks and E13.5 male and female gonads. ATRX is enriched at pericentric heterochromatin of germ and somatic cells in all stages examined. At E11.5, ATRX levels seem to be higher in PGCs compared to the surrounding somatic cells, while at E10.5 and E13.5, the levels of ATRX are comparable between germ cell and somatic nuclei. E10.5 and E11.5 germ cells were identified by OCT4 (*red*) and E13.5 germ cells with TRA98 (*red*). Three or more embryo trunks or gonads were pooled for the nuclear spread preparations and 20–30 PGC, and somatic nuclei were recorded. Representative germ cells are marked with *yellow dashed circles*. *Scale bars* represent 10 μm. **b** Quantification analysis of ATRX levels in the whole nuclear area (N.A.) and pericentric heterochromatin regions (PHC) in the different embryonic stages examined. The levels of ATRX at E11.5 pericentric heterochromatin are significantly increased compared to the soma. *Asterisks* (* or **) indicate significant differences

The α-thalassaemia mental retardation X-linked protein ATRX is a chromatin remodeler and a prominent marker of pericentric heterochromatin in somatic cells, in the mouse zygote, and in neonatal spermatogonia [29, 30, 35, 36]. We explored the presence of ATRX in developing germ cells, only in nuclear spread preparations, since we observed that this protocol yielded the most reproducible and quantifiable results. At E10.5, the levels of ATRX in germ cells and somatic cells were similar (Fig. 8). Interestingly, in the germ cells of E11.5, ATRX immunostaining was increased in pericentric heterochromatin compared to that of the soma. At E13.5, ATRX levels were again comparable in gonadal somatic cells and PGCs (Fig. 8). We did not observe any relocalization of ATRX to the nuclear periphery in any of the E11.5 PGCs examined, in contrast to what was previously reported [27].

### Spatial organization of constitutive heterochromatin in germ cells

**Fig. 9:**
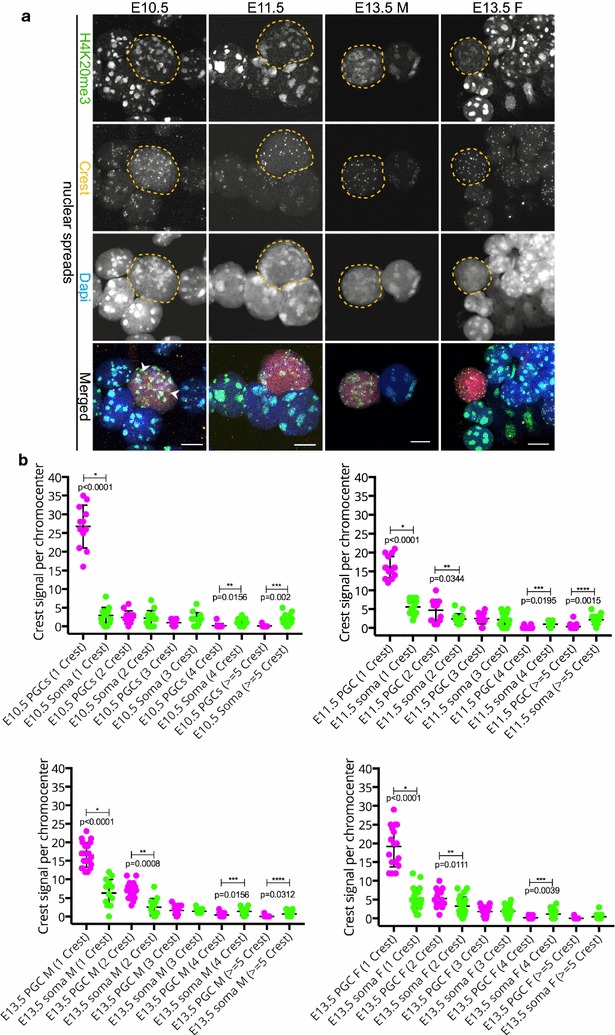
Pericentromeres are more frequently found as single units in PGCs compared to the somatic cells. **a** Nuclear spread preparations were immunostained for CREST (*yellow*) and H4K20me3 (*green*). At E10.5, many small-sized chromocenters [identified by DAPI (*blue*) density and enrichment of H4K20me3) and their corresponding CREST signals can be observed in the PGCs (E10.5 and E11.5 germ cells were identified by OCT4 (*red*) and E13.5 germ cells with TRA98 (*red*)]. Examples of pericentric regions containing a single CREST focus are indicated by *arrowheads*. This pattern of dispersed pericentric heterochromatin organization in germ cells, as opposed to the clustering of pericentric heterochromatin regions and associated centromeres in somatic cells, is observed at least until E13.5. Representative germ cells are marked with *yellow dashed circles*. Three or more embryo trunks or gonads were pooled for the nuclear spread preparations and 20–30 PGC, and somatic nuclei were recorded. *Scale bars* represent 10 μm. **b** Frequency distribution of the number of pericentric heterochromatin regions (defined by delineation of the H4K20me4 areas) containing a specific number of CREST signals as indicated on the *X* axis. *Asterisks* (*, **, *** or ****) indicate significant differences

In order to examine the organization of the chromocenters during germ cell development more globally, we stained nuclear spreads of all developmental stages examined within this study with CREST antisera, a marker of chromosome centromeres. Additionally, we used H4K20me3 to visualize the pericentric heterochromatin. In somatic cells, the chromocenters consisted of more than one pericentric domain, as indicated by the multiple CREST signals within the DAPI-dense (or H4K20me3 enriched) regions (Fig. 9a). This organization reflects the clustering of groups of pericentromeres, which is a common hallmark of chromocenters [8]. However, already at E10.5, we observed a large number of individual pericentric regions, containing only a single CREST signal, within the nucleus of germ cells (Fig. 9a, examples indicated by arrowheads). Counting the number of CREST signals detected per H4K20me3-positive pericentric heterochromatin area, and subsequent analyses of the frequency distribution of pericentric heterochromatin areas containing 1, 2, 3, 4, or more CREST signals revealed that pericentric heterochromatin areas with a single CREST signal are more frequently observed in PGCs compared to somatic nuclei. Conversely, pericentric heterochromatin areas with 4 or more CREST signals are more frequent in the somatic nuclei, at all stages examined (Fig. 9b). This observation may explain the size, number, and intensity differences between the DAPI-dense regions in germ cell versus somatic cell nuclei. Thus, we demonstrate that the organization of the chromocenters in E10.5–E13.5 PGCs is different compared to that of the somatic surrounding cells. A summary of all immunostainings of pericentric heterochromatin markers is presented in Additional file 4.

### Major satellites are not transcribed in E11.5 murine PGCs

**Fig. 10:**
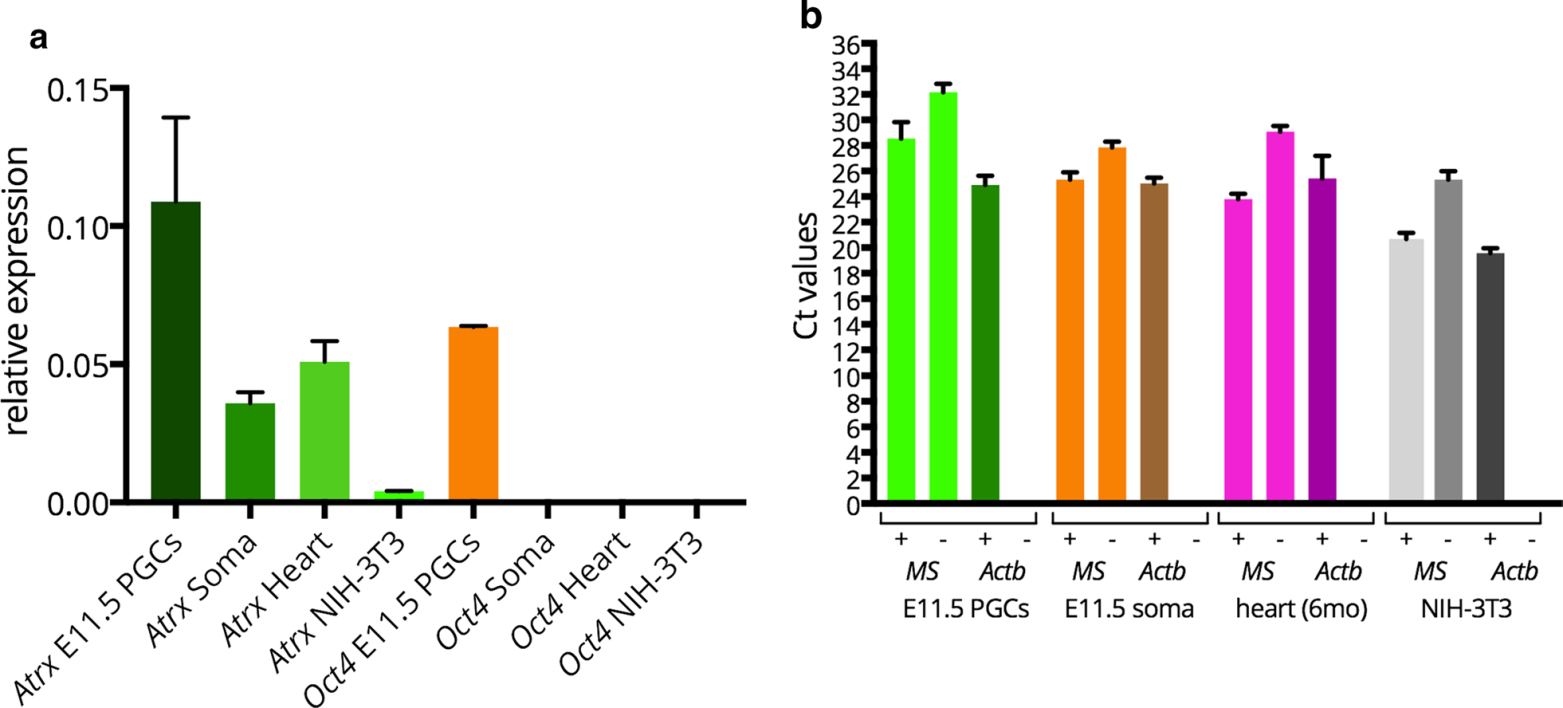
*Atrx* is more highly expressed in E11.5 PGCs compared to the soma, and low major satellite transcription is detected in both cell fractions. **a** Normalized expression values derived from qRT-PCR of E11.5 sorted OCT4-GFP-positive (E11.5 PGCs) and negative (E11.5 Soma) cells, show that expression of *Atrx* and *Oct4* is higher in PGCs compared to the soma, heart tissue, or NIH-3T3 cells. **b** Ct values obtained after qRT-PCR [Ct values of + or − RT reactions are shown, whereby no Ct value was obtained for the –RT performed with the *Actin (Actb)* primers] of major satellites (MS) and *Actb* in OCT4-GFP-positive (E11.5 PGCs) and negative (E11.5 Soma) cells, heart tissue, and NIH-3T3 cells. The difference between + and − RT reaction is a bit smaller for the E11.5 fractions compared to heart and NIH3T3 cells. *Error bars* represent standard deviations of two experiments

In order to examine whether the altered organization of the chromocenters during PGC development is associated with reduced transcriptional repression of major satellites, we FACS-sorted PGCs and somatic cells from developing gonads isolated from E11.5 embryos carrying a transgene-encoding GFP under the control of the *Oct4* promoter. We isolated RNA and analysed mRNA expression in the FACs-sorted cell fractions, heart tissue, and NIH3T3 cells (Fig. 10). From the results of this analysis (Fig. 10a), we conclude that there is a clear enrichment of *Oct4* and *Atrx* mRNA in the E11.5 PGC fraction, in accordance with our immunocytochemical observations. A low level of major satellite transcription is detected in the E11.5 PGCs as well as in the soma, as can be inferred from a similar difference between the + and − RT samples of both fractions. In heart and NIH3T3 cells, the level of major satellite transcription is somewhat higher. This result indicates that there is no increase in transcription of major satellites in PGCs compared to the somatic fraction (Fig. 10b).

## Discussion

At the time of their specification, PGCs are epigenetically identical to the surrounding epiblast and therefore primed towards a somatic fate [37, 38]. In order to activate their germ cell transcriptional network, and at the same time repress their somatic fate, PGCs go through a series of extensive reprogramming events, which have been thoroughly characterized. The reprogramming encompasses DNA demethylation at several genomic loci, including the imprinted genes, but also involves changes in histone modifications [25, 26, 37, 39]. An additional reprogramming cycle has been reported to take place specifically at E11.5, when many histone modifications are transiently lost, including those marking constitutive heterochromatin and its readers [27]. In our study, we carefully re-evaluated epigenetic remodelling targeting specifically constitutive heterochromatin, from the period when PGCs enter the genital ridges (E10.5) [40], until E13.5, when female germ cells enter meiosis, while male germ cells continue to be mitotically active (until E15.5, when they enter a mitotically quiescent phase) [41]. Taking into account that epitope availability can be compromised under certain fixation conditions, we decided to test different preparation and fixation protocols. Indeed, when using our regular fixation protocol in sections, we observed loss of constitutive heterochromatin marks such as H3K9me3, H4K20me3, and HP1α exclusively in the germ cell nuclei, but not in the somatic nuclei, at E11.5. In striking contrast, upon extended fixation in sections, and in nuclear spread preparations, loss of these marks could not be reproduced. We obtained the most consistent results using nuclear spread preparations. This type of single cell methodology may in this case be superior to the former two, due to a better penetrance of the fixative and/or of the antibodies into the spread chromatin [19, 42]. In addition, loss of proteins that localize to the nucleoplasm and cytoplasm, and loss of proteins that are loosely associated with chromatin, may reduce the background signals, when histone modifications are studied. Previous studies used cytospin preparations to examine reprogramming taking place in germ cells [27]. The discrepancy between our results using nuclear spreads and these results using whole fixed cells may thus be attributed to higher background signals or reduced epitope accessibility in a more three-dimensional environment, whereby structural cellular and nuclear components such as membrane and matrix are still present. In support of our results, previous studies [26] could also not reproduce chromatin changes of H1 linker histone or loss of H3K27me3 at E11.5 reported elsewhere [27]. This again illustrates that testing different experimental methodologies is important in order to correctly understand and characterize epigenetic phenomena during different developmental states. In addition, somite counting at these early stages of development is a prerequisite for consistent developmental staging of the different embryos examined, to reduce inter-individual variability and thus improve reproducibility. From our quantification analysis, we can conclude that the level of H3K9me3 at pericentric heterochromatin is transiently reduced in PGCs at E11.5, when ATRX is most increased in these areas, compared to the patterns in surrounding somatic cells. HP1α and HP1β are even more clearly reduced in the pericentric heterochromatin of germ cells compared to somatic cells at (almost) all stages that were studied, but this appears to be compensated, at least in part, by a relative increase of HP1γ. Finally, H4K20me3 is also clearly reduced in pericentric heterochromatin of germ cells compared to surrounding somatic cells between E10.5 and E13.5. It should be taken into account that the differences in level intensities for HP1 isoforms and ATRX between PGCs and somatic cells may be somewhat over- or underestimated, since differences in chromatin structure may also result in differential binding of these proteins to chromatin, which may affect the degree of retention during the nuclear spreading procedure. This issue does not apply to the histone modifications we examined, as they are tightly associated with the DNA. For ATRX, we could confirm the enrichment in PGCs by quantitative RT-PCR. However, for HP1γ in particular, the relative enrichment of this protein in the pericentric heterochromatin of PGCs is more outspoken in the nuclear spreads compared to the sections. Still, most importantly, none of the analysed markers were completely lost at any of the examined stages of development in mouse PGCs.

In light of our observations, it would also be interesting to re-examine whether H3K64me3, a newly identified histone modification marking constitutive heterochromatin, is truly absent from E12.0 to E13.5 germ cells as has been reported [43]. For this immunolocalization study, cryosections of embryo trunks and gonads were stained, using a protocol very similar to our own regular fixation protocol [43]. As our results suggest, such a protocol may not be suitable for answering constitutive heterochromatin localization questions, since epitopes may be masked.

Interestingly, our results show that ATRX, a chromatin remodeler and crucial factor for heterochromatin formation [29, 44], is maintained on pericentric heterochromatin throughout germ cell development. In addition, ATRX is enhanced in these locations of E11.5 PGCs compared to the surrounding somatic cells. Importantly, ATRX has been reported to transcriptionally block expression of major satellites from the maternal genome in the mouse zygote [36]. At this stage, in the early zygote, the maternal pericentromeres are labelled with the classical somatic histone modifications, while these marks are absent from the paternal genome, where transcription of major satellites has been recorded [13]. In addition, studies in embryonic stem cells report that ATRX, together with the histone chaperone DAXX, safeguards the genome against expression of tandem repeats, even when DNA methylation levels are absent from those regions [45]. It would be interesting to examine whether ATRX also performs such a repressive function in PGCs. An additional repressive mechanism against expression of those repeats could involve the formation of 5-hydroxyl-methylated DNA at pericentromeres in PGCs, gradually replacing 5-methyl-cytosine during PGC reprogramming [46]. Therefore, it is possible that more than one mechanism exists for silencing major satellite transcription in PGCs. In our study, we observed a similar low level of major satellite transcription in E11.5 PGCs and somatic cells. Thus, the differences that we observed in pericentric heterochromatin chromatin modification between PGCs and surrounding somatic cells, are not accompanied by a burst of major satellite transcription in PGCs as has been observed in the mouse pre-implantation embryos [22]. This is consistent with the fact that we observed that the vital players of constitutive heterochromatin are continuously present and that ATRX is enriched at pericentric repeats of E11.5 PGCs.

Nevertheless, analyses of the general distribution pattern of centromeres and adjacent pericentric heterochromatin revealed that there is a different organization of constitutive heterochromatin in germ cells compared to the surrounding somatic cells. Specifically, the somatic pericentromere organization into large chromocenters is much reduced in germ cells, where pericentromeres are mainly found as individual units or organized into small chromocenters. We have not identified the cause or the consequence of such an altered pericentromere organization, but this organization may be a natural consequence of germ cell development as they move from a somatic fate towards the more stem cell-like fate of a primordial germ cell and eventually towards the gonocyte. A similar phenomenon of a more dispersed constitutive heterochromatin has been described to take place upon reprogramming of mouse embryonic fibroblasts towards induced pluripotent stem cells, but also in the Nanog-positive cells of the inner cell mass of developing blastocysts [47]. In addition, DAPI-rich regions appear to spread upon induction of embryonic stem cells towards 2-cell stage-like cells [48]. Conversely, when cells differentiate, chromocenters appear to cluster. For example, when male germ cells reach their ultimate differentiated state in mouse adult testes, all chromocenters fuse into a single chromocenter in the nucleus of round, elongating, and condensed spermatids [12]. In addition, differentiation of myoblasts towards myocytes is also accompanied by centromere clustering and chromocenter formation, as well as further enrichment of H3K9me3 and H4K20me3. This differentiation is accompanied with transcriptional activation of major and minor satellite repeats [11].

## Conclusions

The present study reveals that pericentric heterochromatin organization in the embryonic PGC nucleus has changed dramatically from a clustered pattern into individual distribution, but the known hallmarks of heterochromatin are still present. In addition, ATRX, in combination with other mechanisms, may provide an extra level of protection against expression of major satellite transcripts. The observed changes in pericentric chromatin organization could be related to the transition of the germ cells from a somatic fate towards a stem cell-like one.

## Methods

### Collection of mouse embryos for immunofluorescence and immunocytochemistry

Female DBA2 mice were mated with C57BL/6 males to produce F1 fetuses. Mating was confirmed the next morning by the presence of a vaginal plug and recorded as E0.5. At E10.5, E11.5, and E13.5, embryos were dissected out of the uteri and were assessed for somite counting. We scored embryos with 34–36 somites as E10.5 and 44–47 somites as E11.5. We could not determine with precision the somite number at E13.5 (60–62 somites), due to the advanced developmental stage of the embryo. Embryos were kept in ice-cold PBS at all times, before any further processing.

### Tissue processing for immunofluorescence and immunocytochemistry

After embryo isolation from the uteri, embryo regions containing the developing germ cells were dissected from E10.5 to E11.5 embryos. Gonads were isolated from the E13.5 embryos, and the sex was determined by morphology. E10.5 and E11.5 gonadal regions were fixed in ice-cold 4% PFA for 2 h and 3 h, respectively, followed by consecutive washes in PBS. Gonads were fixed for 1.5 h in ice-cold 4% PFA. Tissues were then processed for OCT or paraffin embedding using standard histology procedures. Cryo- and paraffin sections were 10 and 5 μm, respectively.

For the regular and extended fixation, sections were fixed for an additional 10 min at room temperature or for 30 min at 37 °C, respectively, followed by brief PBS washes. The fixation step was performed after the OCT or paraffin was removed from the sections.

### Drying-down or nuclear spread preparations of germ cells

Embryo trunks containing the germ cells from E10.5, E11.5 and gonads from E13.5 embryos were dissected, pooled as indicated in figure legends, and incubated in 500 μl TrypLE™ Express (Thermo Fisher Scientific) for 6 min at 37 °C. Dissociation was followed by two washes with 5% FBS in PBS. Spreads of nuclei for immunocytochemistry were obtained as described by [28].

### Quantification analysis of immunocytochemistry

Single plane images at optimal focus were acquired with a Zeiss LSM 700 microscope (Carl Zeiss, Jena) with the same exposure time for each nucleus of the same stage. A homemade ImageJ macro was used to measure the mean fluorescence intensity of each pericentric heterochromatin marker used for immunofluorescence, in the whole nucleus (defined by the DAPI-positive area), or in pericentric heterochromatin regions, defined by the area that contained signal above the set background threshold for the corresponding marker, and corresponding to regions more strongly stained with DAPI.

### Immunohistochemistry and immunocytochemistry

Heat-mediated (900 W in a microwave for 20 min) epitope retrieval in citrate buffer pH = 6 was performed on paraffin sections. The following staining protocol was performed in all samples. Sections and nuclear spreads were blocked with 2% BSA, 5% donkey serum in PBS (blocking solution) for 30 min at room temperature, followed by primary antibody incubation, diluted in blocking solution, at 4 °C overnight in a humid chamber. The next day, slides were washed in PBS (3 × 5 min) and blocked with secondary antibodies, diluted in blocking buffer, for 1 h at room temperature, in a humid chamber. Slides were then washed in PBS (3 × 5 min) and mounted with ProLong^®^ Gold Antifade Mountant with DAPI (Thermo Fisher Scientific). Confocal imaging was performed on a Zeiss LSM700 microscope (Carl Zeiss, Jena). In this study, the following primary antibodies were used: goat anti-OCT3/4 (N-19) by Santa Cruz (sc-8628) diluted 1:800 for sections and 1:50 for spread preparations, rabbit anti-OCT4 by Abcam (ab19857) diluted 1:250 for sections and 1:50 for spread preparations, rat anti-TRA98 by Abcam (ab82527, 1:500), rabbit anti-DDX4/MVH by Abcam (ab13840, 1:300), anti-rabbit H3K9me3 by Abcam (ab8898, 1:300), rabbit anti-H4K20me3 diluted 1:300 [49], goat anti-HP1α by Abcam (ab77256) diluted 1:200 for sections and 1:400 for spread preparations, mouse anti-HP1β by Abcam (ab10478, 1:200), rabbit anti-HP1γ by Abcam (ab10480, 1:200), and rabbit anti-ATRX (H-300) by Santa Cruz (sc-15408) 1:250, human anti-CREST (CS-1058) by Cortex Biochem 1:1000. The following Alexa Fluor secondary antibodies were used: donkey anti-goat 555/488, donkey anti-rat 555, donkey anti-mouse 488, and donkey anti-rabbit 488 by Thermo Fisher Scientific. All the Alexa Fluor 555 antibodies were used at a dilution of 1:400, while the Alexa Fluor 488 antibodies were diluted 1:250. To detect CREST, we used donkey anti-human 488 DyLight 488 (SA5-10126) by Thermo Fisher Scientific at a 1:250 dilution.

### FACS sorting

Female DBA2 mice were mated with OCT4-GOF18/GFP C57BL/6 males to produce F1 fetuses carrying the OCT4-GFP transgene [50]. Staging of the embryos and dissociation of the tissue were performed as described above (*Drying-down or nuclear spread preparations of germ cells* section). Equal numbers of PGCs and somatic cells were isolated using the SORP-FACSAria II flow cytometer (BD). In more detail, 600 cells per cell population were sorted at 4 °C in 40ul lysis buffer (AM1722, Cells-to-cDNA™ II Kit, Thermo Fisher Scientific) in a 96-well plate containing additionally 2U/μl RNAseOUT (10777-019, Invitrogen). Thereafter, the lysis buffer containing the cells was split into four tubes (two for +RT and two for –RT experiments) and cDNA reactions were performed as described below (RT-qPCR). For NIH-3T3 and heart tissue, RNA was isolated with TRIzol reagent (15596026, Thermo Fisher Scientific). Thereafter, RNA was treated with Turbo DNAse (AM2238, Thermo Fisher Scientific) according to the manufacturer’s instructions.

### RT-qPCR

For quantitative RT-PCR (RT-qPCR), the lysis buffer (Cells-to-cDNA™ II kit, Thermo Fisher Scientific; AM1722) containing the cells was processed for cDNA according to the manufacturer’s instructions. cDNA from NIH-3T3 cells and heart tissue was made with Superscript III (18080093, Thermo Fisher Scientific) according to the manufacturer’s instructions.

All samples were analysed in triplicate in a 15-μl final reaction volume using the BioRad CFX 384 Real-time System. Each reaction contained LightCycler^®^ 480 SYBR Green I Master (04887352001; Roche), primers to a final concentration of 0,2 μM and 1 μl of cDNA. The following primers were used: *Oct4* Fw CCCCAATGCCGTGAAGTTG and Rv TCAGCAGCTTGGCAAACTGTT, *major satellites* Fw GGCGAGAAAACTGAAAATCACG and Rv AGGTCCTTCAGTGTGCATTTC [51], *Atrx* Fw GAGCTTGACGTGAAACGAAGAG and Rv TTGTTGCTGTTGCTGCTGAG, *Actin* Fw ACTATTGGCAACGAGCGGTTC and Rv AGAGGTCTTTACGGATGTCAACG.

After an initial hold at 94 °C for 4 min, reaction mixtures underwent 40 cycles of 30 s at 94 °C, 30 s at 60 °C, and 30 s at 72 °C. Gene expression levels were normalized over *Actin* expression according to the 2-ΔCt method. For the major satellites, the Ct values were compared.

## Additional files

**Additional file 1.** Immunofluorescent analysis of HP1α in paraffin sections using regular and extended fixation protocols. **A** In E10.5 embryos, HP1α (*green*) is enriched at pericentric heterochromatin, but its levels are lower in PGCs compared to somatic cells. From E11.5 onwards, HP1α signal is depleted from DAPI (*blue*)-dense regions in PGCs. **B** Results were similar to **A** when using extended fixation conditions. However, at E11.5 some PGCs could be detected with some signal of HP1α still present at the pericentric heterochromatin (marked by *arrowhead*). Note that in E13.5 male gonads HP1α could not be reproducibly detected in somatic cells, in both **A** and **B**. For each stage, two embryos were analysed per fixation protocol and at least 20 nuclei were recorded. E10.5 and E11.5 PGCs were marked with OCT4 (*red*). E13.5 male and female germ cells were identified by the presence of DDX4/MVH (*red*). Representative images are shown with germ cells highlighted by *dashed yellow circles,* and *scale bars* represent 5 μm.

**Additional file 2.** Immunofluorescent analysis of HP1β in paraffin sections using regular and extended fixation protocols. **A** Using the regular fixation protocol, HP1β signal is enriched at DAPI (*blue*)-dense regions of E10.5 and E11.5 PGCs and somatic cells. HP1β is then substantially reduced in E13.5 female and male germ cells. **B** With the extended fixation protocol, HP1β signal is retained in pericentric heterochromatin of PGCs throughout development. For each stage, two embryos were analysed per fixation protocol and at least 20 nuclei were recorded. E10.5 and E11.5 PGCs were marked with OCT4 (*red*). E13.5 male and female germ cells were identified by the presence of TRA98 (*red*). Representative images are shown with germ cells highlighted by *dashed yellow circles,* and *scale bars* represent 5 μm.

**Additional file 3.** Immunofluorescent analysis of HP1γ in paraffin sections using regular and extended fixation protocols. **A** HP1γ (*green*) signal is enriched at DAPI (*blue*)-dense regions of E10.5 and E11.5 PGCs and somatic cells using the regular fixation protocol. Thereafter, at E13.5, HP1γ could not be detected in male germ cells, while it was still present in E13.5 female germ cell nuclei. **B** Upon application of the extended fixation protocol, enrichment of HP1γ signal was observed in pericentric heterochromatin of E10.5 and E11.5 PGCs. Similar to **A**, HP1γ could not be detected at pericentric heterochromatin of male E13.5 germ cells, while it was still present in E13.5 female germ cells. Note that in both protocols (**A**, **B**) HP1γ could not reproducibly be detected in pericentric heterochromatin of E13.5 somatic cells. For each stage, two embryos were analysed per fixation protocol and at least 20 PGC nuclei were recorded. E10.5 and E11.5 PGCs were marked with OCT4 (*red*). E13.5 male and female germ cells were identified by the presence of TRA98 (*red*). Representative images are shown with germ cells highlighted by *dashed yellow circles,* and *scale bars* represent 5 μm.

**Additional file 4.** Summary of the immunosignals at pericentric heterochromatin of PGCs. The table displays whether a certain histone modification or chromatin-binding protein at the pericentric heterochromatin is detected (+), not detected (−) or detected in some but not all (*) nuclei of PGCs at the embryonic stages indicated. Differences in the degree of enrichment between the pericentric heterochromatin of PGCs and somatic cells are only taken into account in the last column, whereby less enrichment or more enrichment at pericentromeric heterochromatin in PGCs compared to the soma is indicated as <or>, respectively. n.d.: not determined.

## Abbreviations

DAPI: 4′,6-diamidino-2-phenylindole
E11.5: embryonic day 11.5
HP1: heterochromatin protein 1
PGC: primordial germ cell
PRC1 and PRC2: polycomb repressor complexes 1 and 2
RT-qPCR: reverse transcriptase-quantitative polymerase chain reaction
