# Supplementary figures and images for: Silencing markers are retained on pericentric heterochromatin during murine primordial germ cell development
Source: Epigenetics Chromatin. 2017 Mar 11;10:11. doi: 10.1186/s13072-017-0119-3 (PMC5346203; doi:10.1186/s13072-017-0119-3)

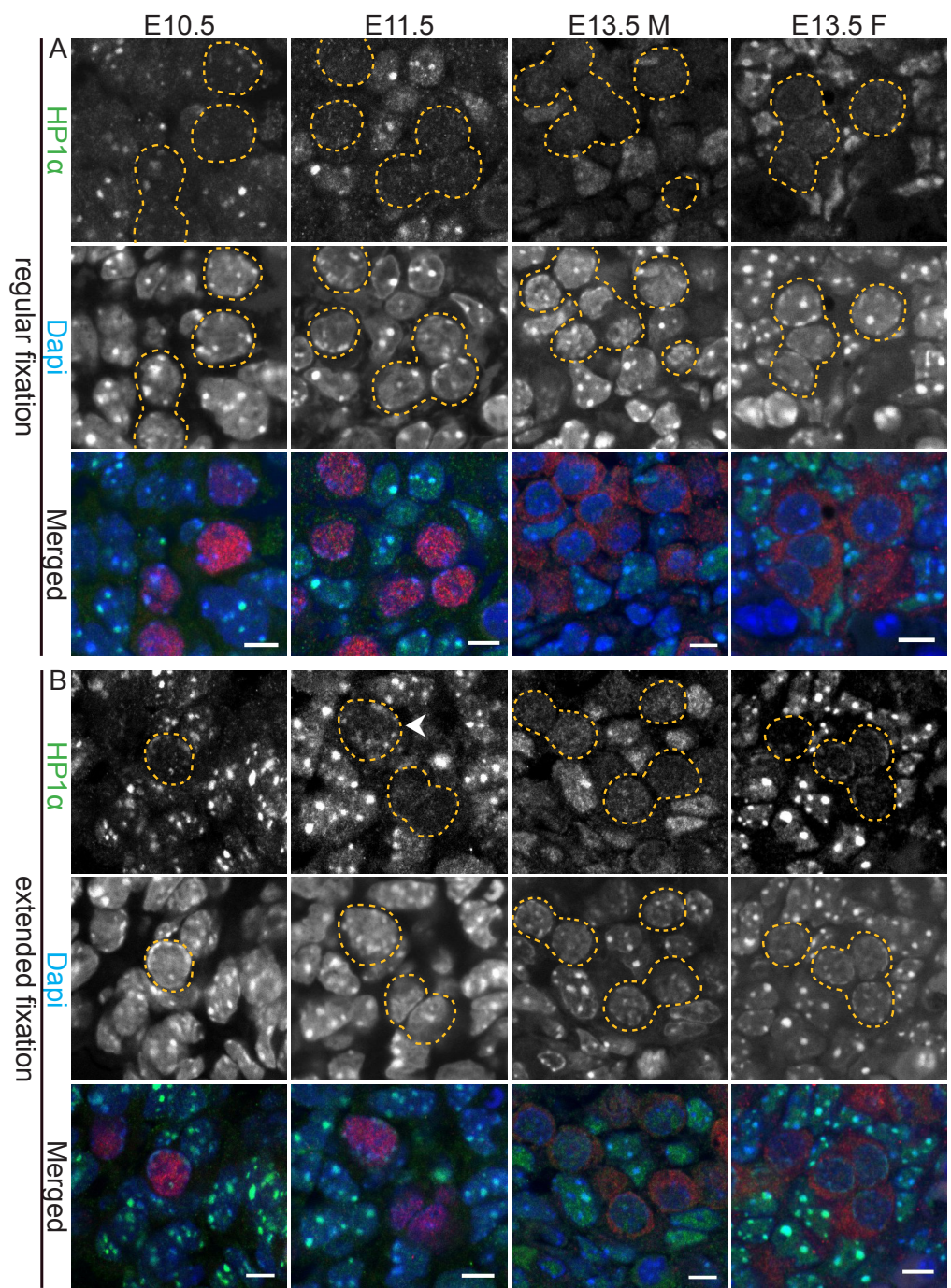

Supplement: Supplementary file 1 — Additional file 1. Immunofluorescent analysis of HP1α in paraffin sections using regular and extended fixation protocols. A In E10.5 embryos, HP1α (green) is enriched at pericentric heterochromatin, but its levels are lower in PGCs compared to somatic cells. From E11.5 onwards, HP1α signal is depleted from DAPI (blue)-dense regions in PGCs. B Results were similar to A when using extended fixation conditions. However, at E11.5 some PGCs could be detected with some signal of HP1α still present at the pericentric heterochromatin (marked by arrowhead). Note that in E13.5 male gonads HP1α could not be reproducibly detected in somatic cells, in both A and B. For each stage, two embryos were analysed per fixation protocol and at least 20 nuclei were recorded. E10.5 and E11.5 PGCs were marked with OCT4 (red). E13.5 male and female germ cells were identified by the presence of DDX4/MVH (red). Representative images are shown with germ cells highlighted by dashed yellow circles, and scale bars represent 5 μm. [file 13072_2017_119_MOESM1_ESM.pdf]

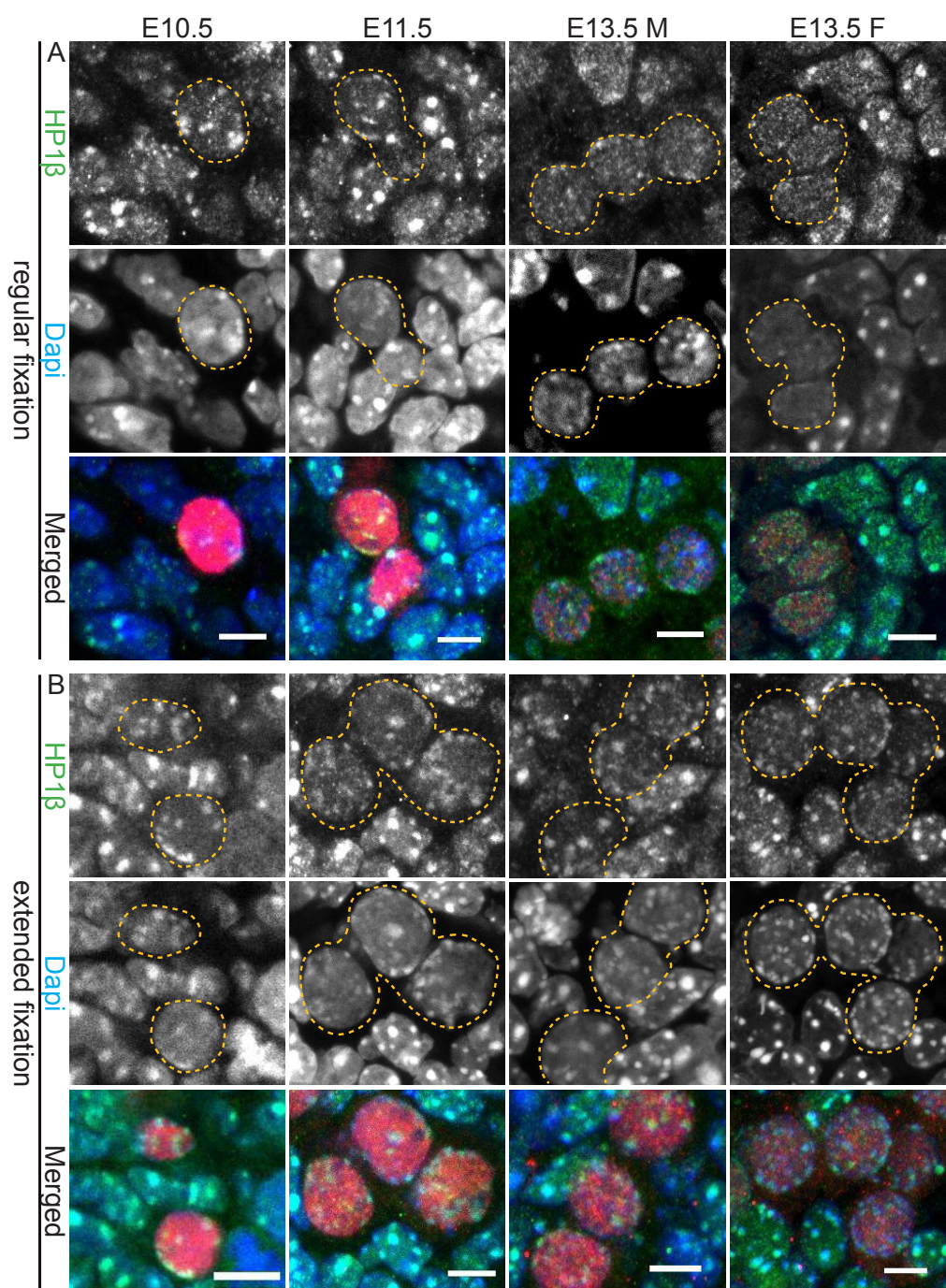

Supplement: Supplementary file 2 — Additional file 2. Immunofluorescent analysis of HP1β in paraffin sections using regular and extended fixation protocols. A Using the regular fixation protocol, HP1β signal is enriched at DAPI (blue)-dense regions of E10.5 and E11.5 PGCs and somatic cells. HP1β is then substantially reduced in E13.5 female and male germ cells. B With the extended fixation protocol, HP1β signal is retained in pericentric heterochromatin of PGCs throughout development. For each stage, two embryos were analysed per fixation protocol and at least 20 nuclei were recorded. E10.5 and E11.5 PGCs were marked with OCT4 (red). E13.5 male and female germ cells were identified by the presence of TRA98 (red). Representative images are shown with germ cells highlighted by dashed yellow circles, and scale bars represent 5 μm. [file 13072_2017_119_MOESM2_ESM.pdf]

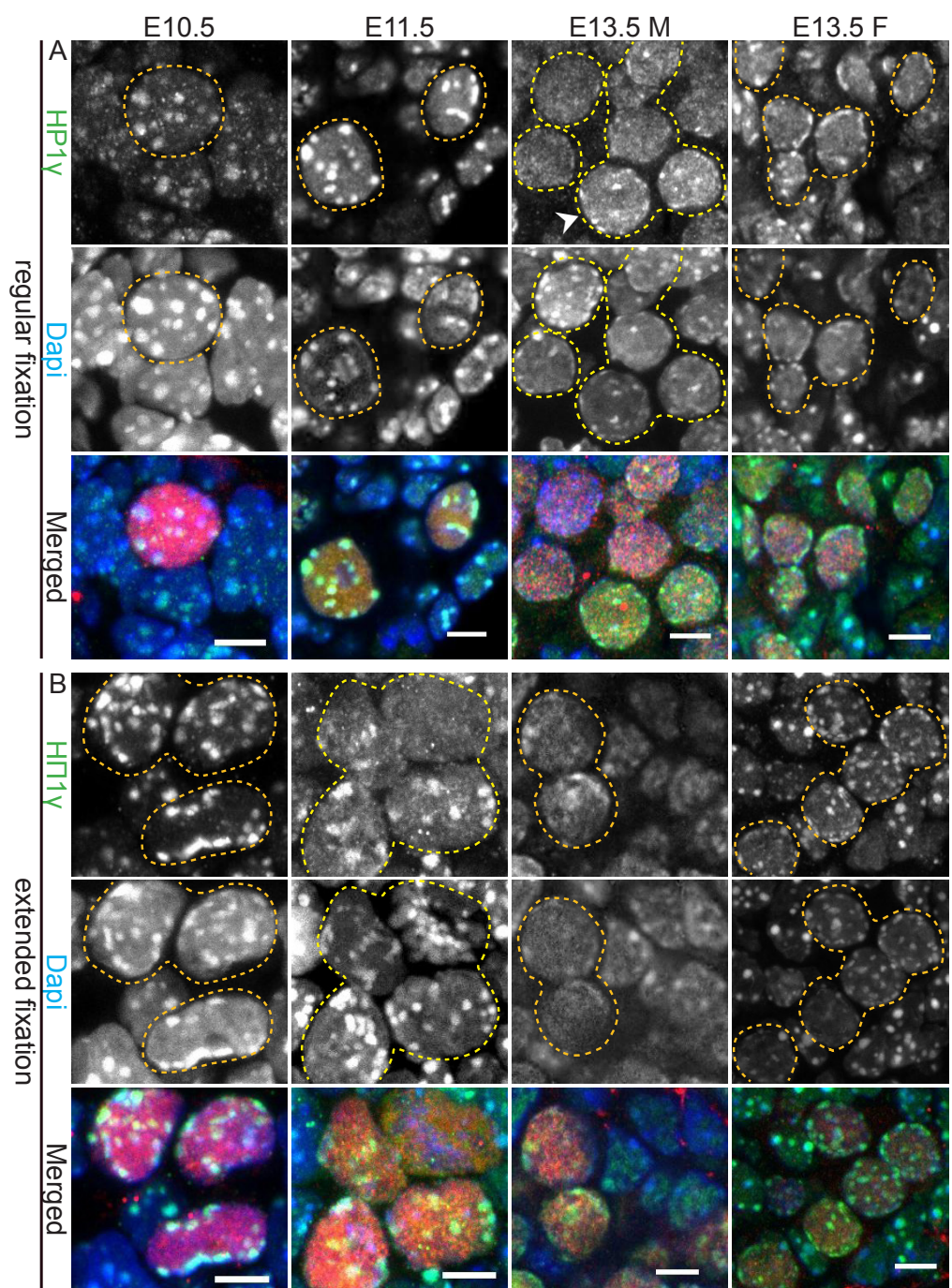

Supplement: Supplementary file 3 — Additional file 3. Immunofluorescent analysis of HP1γ in paraffin sections using regular and extended fixation protocols. A HP1γ (green) signal is enriched at DAPI (blue)-dense regions of E10.5 and E11.5 PGCs and somatic cells using the regular fixation protocol. Thereafter, at E13.5, HP1γ could not be detected in male germ cells, while it was still present in E13.5 female germ cell nuclei. B Upon application of the extended fixation protocol, enrichment of HP1γ signal was observed in pericentric heterochromatin of E10.5 and E11.5 PGCs. Similar to A, HP1γ could not be detected at pericentric heterochromatin of male E13.5 germ cells, while it was still present in E13.5 female germ cells. Note that in both protocols (A, B) HP1γ could not reproducibly be detected in pericentric heterochromatin of E13.5 somatic cells. For each stage, two embryos were analysed per fixation protocol and at least 20 PGC nuclei were recorded. E10.5 and E11.5 PGCs were marked with OCT4 (red). E13.5 male and female germ cells were identified by the presence of TRA98 (red). Representative images are shown with germ cells highlighted by dashed yellow circles, and scale bars represent 5 μm. [file 13072_2017_119_MOESM3_ESM.pdf]
