# Supplementary material for: Silencing markers are retained on pericentric heterochromatin during murine primordial germ cell development
Source: Epigenetics Chromatin. 2017 Mar 11;10:11. doi: 10.1186/s13072-017-0119-3 (PMC5346203; doi:10.1186/s13072-017-0119-3)
Supplement: Supplementary file 4 — Additional file 4. Summary of the immunosignals at pericentric heterochromatin of PGCs. The table displays whether a certain histone modification or chromatin-binding protein at the pericentric heterochromatin is detected (+), not detected (−) or detected in some but not all (*) nuclei of PGCs at the embryonic stages indicated. Differences in the degree of enrichment between the pericentric heterochromatin of PGCs and somatic cells are only taken into account in the last column, whereby less enrichment or more enrichment at pericentromeric heterochromatin in PGCs compared to the soma is indicated as , respectively. n.d.: not determined. [file 13072_2017_119_MOESM4_ESM.pdf]

Table 1: Summary of the immunosignals at pericentric heterochromatin of PGCs

	4% PFA (regular) paraffin	4% PFA (extended) paraffin	4% PFA (regular) cryo	4% PFA (extended) cryo	1% PFA nuclear spreads
**H3K9me3**	No signal	No signal	+E10.5<br>-E11.5<br>+E13.5♂<br>+E13.5♀	+E10.5<br>+E11.5<br>+E13.5♂<br>+E13.5♀	+E10.5<br><E11.5<br>+E13.5♂<br><E13.5♀
**HP1α**	+E10.5<br>-E11.5<br>-E13.5♂<br>-E13.5♀	+E10.5<br>*E11.5<br>-E13.5♂<br>+E13.5♀	n.d.	n.d.	<E10.5<br>+E11.5<br><E13.5♂<br><E13.5♀
**HP1β**	+E10.5<br>+E11.5<br>-E13.5♂<br>-E13.5♀	+E10.5<br>+E11.5<br>+E13.5♂<br>+E13.5♀	n.d.	n.d.	<E10.5<br><E11.5<br><E13.5♂<br><E13.5♀
**HP1γ**	+E10.5<br>+E11.5<br>*E13.5♂<br>+E13.5♀	+E10.5<br>+E11.5<br>+E13.5♂<br>+E13.5♀	n.d.	n.d.	>E10.5<br>>E11.5<br>+E13.5♂<br>+E13.5♀
**H4K20me3**	+E10.5<br>-E11.5<br>*E13.5♂<br>*E13.5♀	+E10.5<br>+E11.5<br>+E13.5♂<br>+E13.5♀	n.d.	n.d.	<E10.5<br><E11.5<br><E13.5♂<br><E13.5♀
**ATRX**	n.d.	n.d.	n.d.	n.d.	+E10.5<br>>E11.5<br>+E13.5♂<br>+E13.5♀
